# Dewatering performance of aerobic granular sludge under centrifugal with different sludge conditioning agent

**DOI:** 10.3389/fmicb.2024.1386557

**Published:** 2024-06-17

**Authors:** Ailan Yan, Yongfei Chen, Ningyu Li, Ting Ma, Yiting Qi, Dong Xu

**Affiliations:** ^1^Nanxun Innovation Institute, Zhejiang University of Water Resources and Electric Power, Hangzhou, China; ^2^College of Water Conservancy and Environmental Engineering, Zhejiang University of Water Resources and Electric Power, Hangzhou, China; ^3^Hangzhou Yongzhan Environment Technology Co., Ltd., Hangzhou, China; ^4^China Railway Eryuan Engineering Group East China Exploration & Design Co., Ltd., Hangzhou, China

**Keywords:** aerobic granular sludge, dewatering performance, dewatering conditioner, alum sludge, dewatering mechanism model

## Abstract

The aerobic granular sludge(AGS) technology draw scientific researchers attention, and more and more scientific research focuses on it, due to its superior advantages, such as good settling performance, high biological phase, high toxicity resistance and multiple biological effects. With the rapid development of AGS technology, a considerable amount of residual AGS will be produced, and dehydration is the biggest bottleneck of sludge reduction. This study investigated the dewatering process and method of residual AGS cultured by continuous flow experiment. Experiments were conducted using centrifugal dewatering technology with a dosing scheme to analyze the granular sludge dewatering process, and investigate the release process of EPS component in AGS dewatering. Our results implied the specific resistance of AGS has a very low value ((1.82 ± 0.03) × 10^9^ m/kg) and it was not obvious for the conditioning effect of chemical conditioner on AGS dewatering. However, the moisture content can be reduced to 63.5% after dewatering with the presence of inorganic substances. The addition of drinking water treatment plant sludge (Alum sludge) can improve the efficiency of the dewatering of AGS. A possible dewatering process of AGS dewatering was proposed which was divided into two stages: First, a considerable amount of free water in the sludge was quickly removed under the action of gravity without pressure filtration. Second, the bound water release required cooperation between applying centrifugal or pressing force to grind granular cells and separate protein-like substances with the inorganic matter inside the granular sludge. The possible mechanism of AGS dewatering and hypothesis dewatering process are useful to optimize the AGS dewatering process.

## Introduction

More and more scientific researchers attach great importance to sewage treatment (Zhou X. et al., 2014; Sarvajith and Yarlagadda, 2021; Erdirencelebi and Ebrahimi, 2022). With the generation of a large amount of urban sewage and the continuous improvement of sewage treatment rate, the harmless treatment rate of urban sludge also needs to be improved accordingly (Mucha and Mikosz, 2021). In the future, the harmless treatment rate of urban sludge needs to reach more than 90%, therefore, it is urgent to solve the difficulties in the treatment and disposal of sewage sludge, achieve harmlessness, and promote resource utilization.

The activated flocculent sludge(AFS) process is the most widely used sewage treatment technology, with more than 85% of urban sewage treatment plants adopting it (Zhou et al., 2023). However, the remaining flocculent sludge has a strong odor, small particle size, high viscosity, and a high water content, usually above 99% (Sharma et al., 2020). If the sludge water content is reduced from 99 to 97%, then the volume of the sludge will decrease by two-thirds (Srivastava et al., 2021). However, dehydration is the biggest bottleneck for sludge reduction (He et al., 2020). Compared to AFS, aerobic granular sludge (AGS) has irreplaceable advantages: a regular shape, compact structure, rapid settlement, mud-water separation, and less residual sludge (Johnson et al., 2020; Rollemberg et al., 2020). It can also achieve simultaneous denitrification and phosphorus removal with multiple bacterial species and has strong resistance to shock loads (Martin and Ii, 2022; Wang et al., 2022). Therefore, AGS technology is considered the “most promising biological sewage treatment technology in the 21st century.” Although it has a short research history of AGS from its first discovery in 1991, the significant achievements and experience have been accumulated in the cultivation, characteristics, and applications of granular sludge over the past 30 years (Lili et al., 2005; Wang et al., 2008; Zhou D. et al., 2014; Chu et al., 2015; Franca et al., 2018). However, there is still relatively little research on the dehydration performance of aerobic granular sludge.

During the dehydration of flocculent sludge, conditioning agents, also known as dehydrating agents, are commonly used to condition the residual sludge before dehydration (Masihi and Badalians Gholikandi, 2020). These agents can be divided into two main categories: inorganic conditioning agents and organic conditioning agents. Inorganic conditioning agents, the most effective, cheapest, and commonly used ones, mainly include iron and aluminum salts (Peeters et al., 2013; Wei-Ran et al., 2017; Weijun et al., 2021). They can significantly accelerate the concentration process of sludge and improve the filtration and dehydration effect. Compared to inorganic conditioning agents, organic conditioning agents require a smaller dosage, typically ranging from 0.1 to 0.5% of the dry solid weight of the sludge, and they are non-corrosive (Luo et al., 2013). Among them, cationic polyacrylamide can neutralize the negative charges on the surface of sludge particles and produce a bridging effect between particles, exhibiting strong cohesion and significant conditioning effects. The type and dosage of conditioning agents vary significantly for different types of sludge.

Extracellular polymeric substances (EPS) are high-molecular-weight compounds secreted by microorganisms, mainly composed of polysaccharides (PS) and proteins (PN), which account for approximately 70–80% of the total EPS (Zhang et al., 2021). Additionally, they also contain small amounts of DNA, humic substances, and lipids (Sheng et al., 2010). Due to the presence of charged functional groups in EPS, the content and composition of EPS in sludge greatly influence its surface charge status. The PN surface carries hydrophilic polar groups such as -COOH, -SH, -NH_4_^+^, -OH, and -CONH_4_, which favor the adsorption of water by the floc. On the other hand, the PS surface carries functional groups such as -COO^−^, SO_4_^2−^, PO_4_^3−^, which tend to form a hydrated film around the bacterial cells. Therefore, there is a close relationship between the characteristics of EPS and the floc structure and dewatering performance of sludge.

In order to enhance public awareness of aerobic granular sludge and expedite its industrial application, further research on its dehydration properties is warranted. This study aims to investigate the dehydration performance of AGS cultivated in a continuous flow system. Different dehydrating agents will be added, and EPS produced during dehydration will be measured to conduct dehydration performance experiments. Ultimately, the goal is to propose a mechanism for the dehydration of aerobic granular sludge.

## Materials and methods

### Reactor design and sludge samples

**Figure 1:**
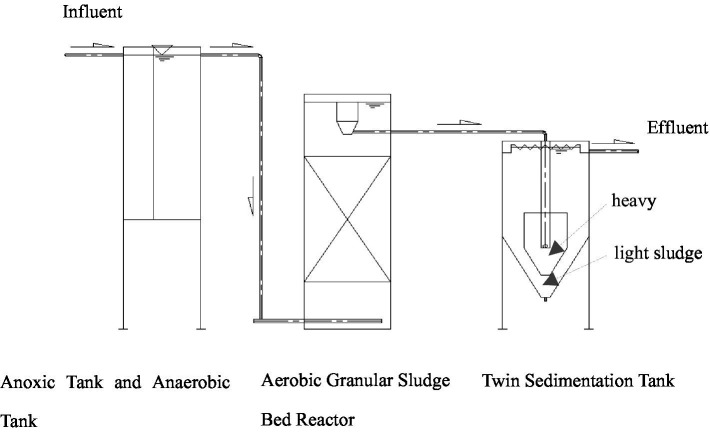
Process flow chart for AGS culturing by continuous flow experiment.

The process for culturing AGS by continuous flow reactor integrated both the mud-membrane coupled biological treatment technology and the twin sedimentation tank sludge screening and separation technology, mainly including anoxic tank and anaerobic tank, AGS Bed Reactor, and twin sedimentation tank (A^2^/O-TST) (Figure 1). The twin sedimentation tank was radially divided into an inner and an outer zone. The heavy sludge (mostly granular sludge) that settled in the inner zone was recirculated, while the light sludge (mostly flocculent sludge) that settled in the outer zone was discharged as excess sludge.

**Figure 2:**
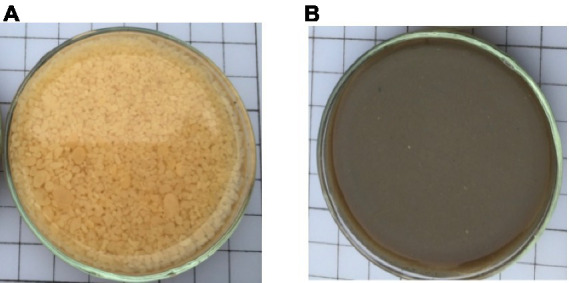
AGS **(A)** from the experiment reactor and AFS **(B)** from the secondary sedimentation tank in a local wastewater treatment plant.

The AGS used in the experimental sample has been cultivated and collected from the continuous flow reactor, independently developed by the team. After culturing for 6 months, the AGS had the size of 1.8 ~ 2.8 mm spherical or oval with orange or yellow for the experiment (Figure 2A). The solid concentration, volume index (SVI), TS, TSS, volatile solids (*VS*), and volatiles suspended solids (VSS) value were measured (APHA, 2005).

The AFS for experiments was taken from the secondary sedimentation tank in a local wastewater treatment plant in China, and the solid concentration was measured (Figure 2B). The moisture content was approximately 99.1%, the pH value was 6.8–7.0 and the VSS/SS was about 75%, which had been added CPAM for dewatering after in WWTP.

The water treatment plant (DWTP) sludge (alum sludge) had a moisture content of 64% which was mechanically reduced on-site. Within the water treatment process, polyaluminum chloride (PACl) was utilized as the primary coagulant for the purification of drinking water, leading to potential deposits of residual PACl and its reaction products within the alum sludge. Additionally, the use of CPAM and surfactants was widespread to enhance the mechanical dewatering of the sludge. 27 g/t PACl and 0.21 g/t CPAM were used in this water treatment plant (WTP). After treatment, the alum sludge exhibited a final moisture content of approximately 64%, a pH range of 6.9–7.1, and a yellowish-brown coloration.

All collected samples were stored at the same conditions and performed duplicates.

### Centrifugal equipment for experimentation

**Figure 3:**
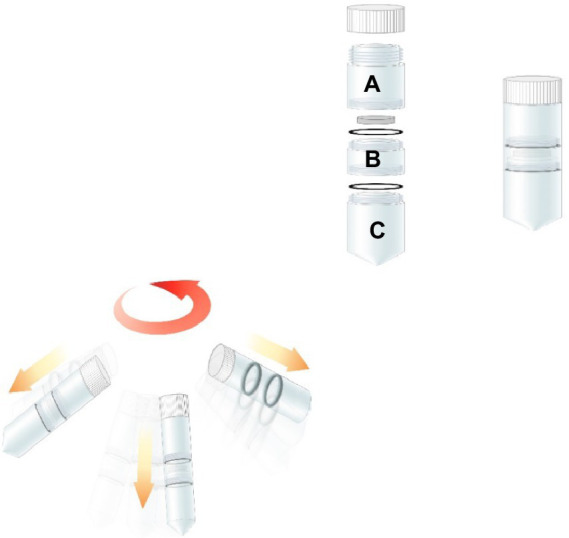
The special centrifugal tube for centrifugal dewatering experiment.

The special centrifugal tube was designed for centrifuge dewatering (Φ30 mm × 115 mm) (Figure 3) using the laboratory low-speed centrifuge with a maximum speed of 4,000 rpm. The Centrifugal tube device was separated into three sections: A was for injection sample, B was for collecting solid with filter-pore and filter cloth and C was for gathering the centrifuged liquid. C.

**Figure 4:**
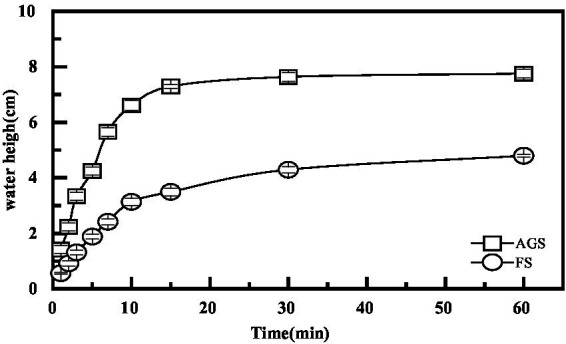
The dynamic between AGS and AFS filtrate over time.

### Sludge conditioning

The sewage treatment plant typically necessitates pretreatment, known as sludge conditioning, before sludge dewatering. This process involves the addition of a flocculant polymer. Among the various types of flocculant polymers, cationic polyacrylamide (CPAM) has proven to be relatively effective in Wastewater treatment plant. Consequently, CPAM was selected as the flocculant polymer for the experiments. These experiments were conducted using various concentration gradients of CPAM, namely 0 g/kg, 20 g/kg, and 40 g/kg, respectively. The necessary reagent was kindly provided by the Wastewater Treatment Plant. (Jiangsu Gaoda Chemical Co., China).

The flocculant polyaluminum chloride (PACl) is widely utilized in drinking water treatment plants, resulting in the generation of significant quantities of alum sludge. This sludge contains numerous inorganic particles and residual aluminum hydroxide. The alum sludge with a moisture content of 64.4% was combined with Activated Granular Sludge (AGS) originating from the experimental twin sedimentation tank. The blending was done at various dry-weight ratios, and the mixture was stirred for 10 min to ensure complete homogenization. The notation “1 kg/kg” indicates a combination of 1 kg of dry alum sludge with 1 kg of dry AGS.

### Specific resistance to filtration

The specific resistance to filtration (SRF) parameter is commonly employed to characterize the filterability of a substrate. It quantifies the resistance encountered by sludge when attempting to drain its water content through a porous filter medium, thus providing valuable insights into the filtration process. The determination of SRF values for sludge samples followed procedures that are consistent with established literature (Yan et al., 2018). This allows for a reliable assessment of the sludge’s filtration properties and provides insights into the effectiveness of solid–liquid separation processes.


$$r=\frac{2PA^{2}b}{\mu w}$$

In the formula, *P* represents the driving force (*N*/m^2^) in the dehydration process; *A* stands for the filtration area (m^2^); μ denotes the viscosity (*N*•S/m^2^) of the filtrate; *W* is the weight (kg/m^3^) of dry sludge generated per unit volume of filtrate; and *b* is a slope coefficient (S/m^6^) determined in the measurement of specific resistance, whose value depends on the nature of the sludge.

### Free water and bound water content

Free water exists independently of solid particles and encompasses void water, which remains unaffected by capillary force. The free water proportion in AGS was determined by a Buchner funnel with a diameter of 12 cm for 30 min at a pressure of 0.05Mpa. Subsequently, the resultant filtered sludge cake moisture content was determined.

### EPS extraction and analysis

AGS samples were centrifuged under various speeds of 0 rpm, 2,000 rpm, and 4,000 rpm for 20 min at 20°C. The supernatant obtained from each centrifugation. Subsequently, the EPS (extracellular polymeric substances) from the sludge was extracted with a modified methanal-NaOH extraction method, following the methodologies described by Jian (2003) and Lemaire et al. (2008). The EPS biopolymers primarily consisted of proteins and polysaccharides, which were measured using distinct methods. Specifically, Kaumas Bradford method was employed to determine the protein (PN) concentration and the phenol sulfuric acid method was used to assay the polysaccharide (PS) content (Clesceri et al., 1992).

### 3D-excitation-emission matrix

The alum sludge cake was wrapped with filter paper, then placed in an oven at 103°C to dry for 6 h. After drying, the mud cake was placed on the sample loading platform after placed in a vacuum evaporation coating instrument for surface spraying with a gold film to enhance the conductivity of the alum sludge.

### Statistical analysis

MS-Excel software has been employed to analyze the acquired datasets and craft the illustrative figures. The data showcased herein represented the averages alongside their corresponding standard deviations. Additionally, the ANOVA test has been used to delve into the disparities among the various measurement sets. The observed variations were deemed statistically significant when evaluated within a 95% confidence interval (*p*-value <0.05).

## Results and discussion

### Dewatering performance of AGS

It was found that the smaller SRF of sludge, the easier it was to dewater. Filtration would be easier when the SRF value was under 0.4 × 10^13 m/kg (Yan et al., 2018), and filtration became challenging if the SRF value fell between (0.5–0.9) × 10^13 m/kg. When the SRF value exceeds 1 × 10^13 m/kg, dewatering would be even more difficult. The SRF value of AGS (1.82 ± 0.03 × 10^9 m/kg), cultured in this continuous flow system, was significantly lower than the SRF value of AFS (3.23 ± 0.03 × 10^14m/kg). This indicated that AGS exhibited much better dewatering performance than AFS.

2.0 g/kg SS CPAM was treated in the AGS, and the SRF value was 1.78 ± 0.03 × 10^9 m/kg, while treated with the same amount of CPAM, the AFS had an SRF value of 1.09 ± 0.08 × 10^14 m/kg. Then the SRF values of AGS and AFS were 1.61 ± 0.05 × 10^9 m/kg, and 1.79 ± 0.08 × 10^13 m/kg (Table 1), respectively, when treated with 40 mg/g SS CPAM. It can be seen that the dehydration of mature granular sludge was attributed to its high specific gravity and large particle size. Without the need for additives, the specific SRF of this sludge fell within the range of easily filterable sludge. The addition of conditioning agents did not significantly alter the SRF, further confirming that granular sludge had good dehydration performance and was easier to dehydrate than ordinary sludge.

**Table 1:** SRF values of AGS and AFS.

	CPAM (g/kg)		
	0	20	40
SRF _(AGS)_ (m/kg)	1.82 ± 0.03 × 10^9	1.78 ± 0.03 × 10^9	1.61 ± 0.05 × 10^9
SRF _(AFS)_ (m/kg)	3.23 ± 0.03 × 10^14	1.09 ± 0.08 × 10^14	1.79 ± 0.08 × 10^13

### The amount of free water

**Figure 5:**
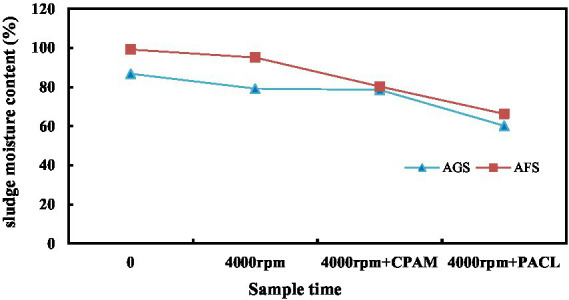
The sludge moisture content variations of AGS and AFS with CPAM and PACl conditioner under 4,000 rpm centrifugal Dewatering.

The filtration rate of AGS through filter paper was significantly faster compared to AFS. Additionally, the filtered fluid volume from AGS increased rapidly during the cylinder filtration process, as demonstrated in Figure 5. The average AGS filtrate volume was 7.31 mL after 15 min, indicating there’s no free water exudation. Conversely, in the AFS funnel, a significant amount of water remained trapped within the sludge, and the dripping speed was considerably slower, resulting in 3.5 mL filtered fluid.

AGS filtered fluid value was 7.75 mL after 1 h of filtration, while it was 4.79 mL for AFS. At the end of the experiment, the sludge samples were dried at 105°C for 6 h, revealing a moisture content of 96.72% for AGS and 98.21% for AFS, respectively. This represents a difference of 1.5% between the two sludge types.

Under identical mass and moisture content conditions, it is evident that AGS exhibits superior velocity and a larger capacity to eliminate free water compared to AFS. The structural formation of AGS modifies the water composition within the sludge mass, subsequently increasing the proportion of free water. Additionally, the larger particle size of AGS in this study (2 ~ 2.5 mm) results in a reduced specific surface area and consequently, a lower adsorption water content. Typically, a smaller sludge structure or particle diameter translates to a higher concentration of colloidal particles within the flocculated sludge, indicating a higher organic matter content. This makes it more challenging to dehydrate the sludge. Conversely, AGS exhibits the opposite trend, with its free water filtration performance surpassing that of AFS. The binding force of free water in AGS is relatively low, allowing for its effortless removal by gravity. This contributes to a reduction in sludge volume, making AGS a more effective and efficient option for water treatment processes.

### Performance of centrifugal dewatering

The binding force between bound water and capillary water (which accounts for approximately 10 to 20% of the moisture content in sludge) with the sludge is relatively strong, and thus requires external force, such as mechanical dehydration, for separation. As shown in Figure 5, the dewaterability of granular sludge is significantly higher than that of flocculent sludge. Before centrifugation, the moisture content of AGS was 86.8%, while that of AFS was 99.2%. After centrifugation at 4000 rpm, the moisture content of AGS was 79.2%, while that of AFS was 95.1%. When 2.0 g/kg of the chemical conditioning agent CPAM was added to condition the sludge before centrifugation at 4000 rpm, the AGS cake moisture content decreased to approximately 78.6%, indicating a slight decrease compared to without CPAM. However, the moisture content of the AFS cake decreased significantly from 95.1 to 80.3%. When mixing granular sludge with water treatment plant sludge at a ratio of 1:1, the moisture content of AGS decreased to approximately 60.2%, while that of AFS decreased to 66.3%. This suggests that the dense internal structure of granular sludge, along with the centrifugal force, allows for mutual squeezing between particles during dehydration, releasing more water from the sludge. The addition of water treatment plant sludge, which contains more inorganic particles, further increases the squeezing pressure between particles, forcing out more interstitial water and improving dewatering performance. Obviously, granular sludge can achieve good dewatering results without the need for CPAM conditioning. The addition of water treatment plant sludge for collaborative dehydration can further enhance the dewatering effect, potentially allowing for reduced CPAM usage.

### Release of EPS during the dewatering process

EPS, as a product of microbial activity, surrounds bacteria like a capsule and continuously secretes viscous polymers into the solution. Although the high water content of extracellular polymers provides good protection for microorganisms, the increased bound water becomes a major obstacle to sludge dehydration, making it difficult to remove more water embedded in the sludge floc by mechanical filtration. Therefore, analyzing the changes in EPS content in the centrifugal fluid during centrifugal dehydration of granular sludge can further assess the dehydration performance of granular sludge.

**Figure 6:**
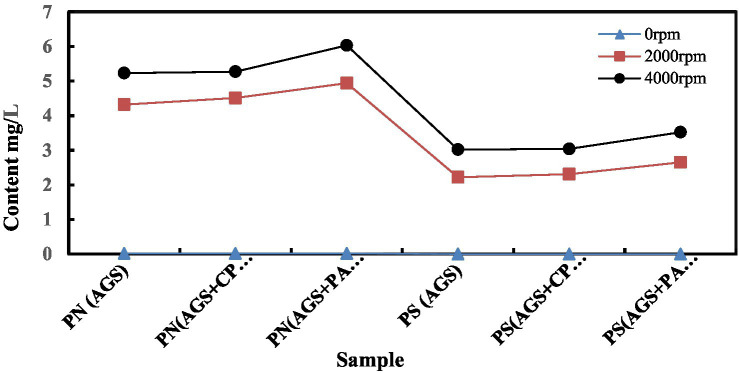
The PN and PS content variations of AGS and AFS with CPAM and PACl conditioner under 2,000 and 4,000 rpm centrifugal Dewatering.

Through the measurement of EPS content in the centrifugal fluid of three types of granular sludge: sample A (AGS), sample B (AGS with 2.0 g/kg CPAM), and sample C (a mixture of AGS and water treatment plant sludge at a ratio of 1:1), the results showed that mechanical centrifugation can cause EPS in the granular sludge to peel off and dissolve (Figure 6). When the original granular sludge was allowed to stand without centrifugation, a small amount of EPS was found in the filtrate of the three types of AGS. Further analysis revealed only a small amount of PN, with a value of 0.02 mg/L, and no PS was detected. When the samples were placed in centrifuge tubes and centrifuged at a speed of 2,000 rpm, the EPS content in the centrifugal fluid of A, B, and C granular sludge increased, with PN values of 4.32 mg/L, 4.51 mg/L, and 4.94 mg/L, respectively, while PS values of 2.22 mg/L, 2.31 mg/L, and 2.65 mg/L, respectively. When the centrifugation speed of A, B, and C granular sludge was increased to 4,000 rpm, the PN values were 5.23 mg/L, 5.27 mg/L, and 6.03 mg/L, respectively, and the PS values were 3.02 mg/L, 3.04 mg/L, and 3.52 mg/L, respectively. It can be seen that adding CPAM conditioning agent to the granular sludge before centrifugation did not significantly affect the PN and PS content in the centrifugal fluid, further indicating that CPAM has a minimal effect on the dehydration conditioning of granular sludge. When adding water treatment plant sludge at a ratio of 1:1, the EPS content in the centrifugal fluid increased significantly, indicating that the water treatment plant sludge increased the grinding between the cells of the granular sludge. Under the action of external forces, the inorganic substances within the structure of the granular sludge rubbed and pressed against each other, causing the cells within the granular sludge to rupture. Cell disruption led to the release of intracellular substances, which facilitated the release of bound water from the granular sludge.

### Speculating on the mechanism model of dewatering of AGS

Generally speaking, the smaller the sludge structure or the smaller the sludge particle diameter, the more floc and colloidal particles are contained in the sludge, that is, the higher the organic matter content, the more difficult it is for the sludge to be dewatered. On the contrary, it is more favorable. Aerobic granular sludge has a high wet density and a relatively low moisture content. From a structural perspective, the advantage of granular sludge dewatering performance compared with ordinary flocculated sludge lies in the fact that the formation of granular sludge changes the water content composition of the sludge mass and increases the proportion of easily removable interstitial water. In addition, mature granular sludge has a larger particle size, with an average particle size of 2 to 2.5 mm, so it has a smaller specific surface area and correspondingly less adsorbed water content.

**Figure 7:**
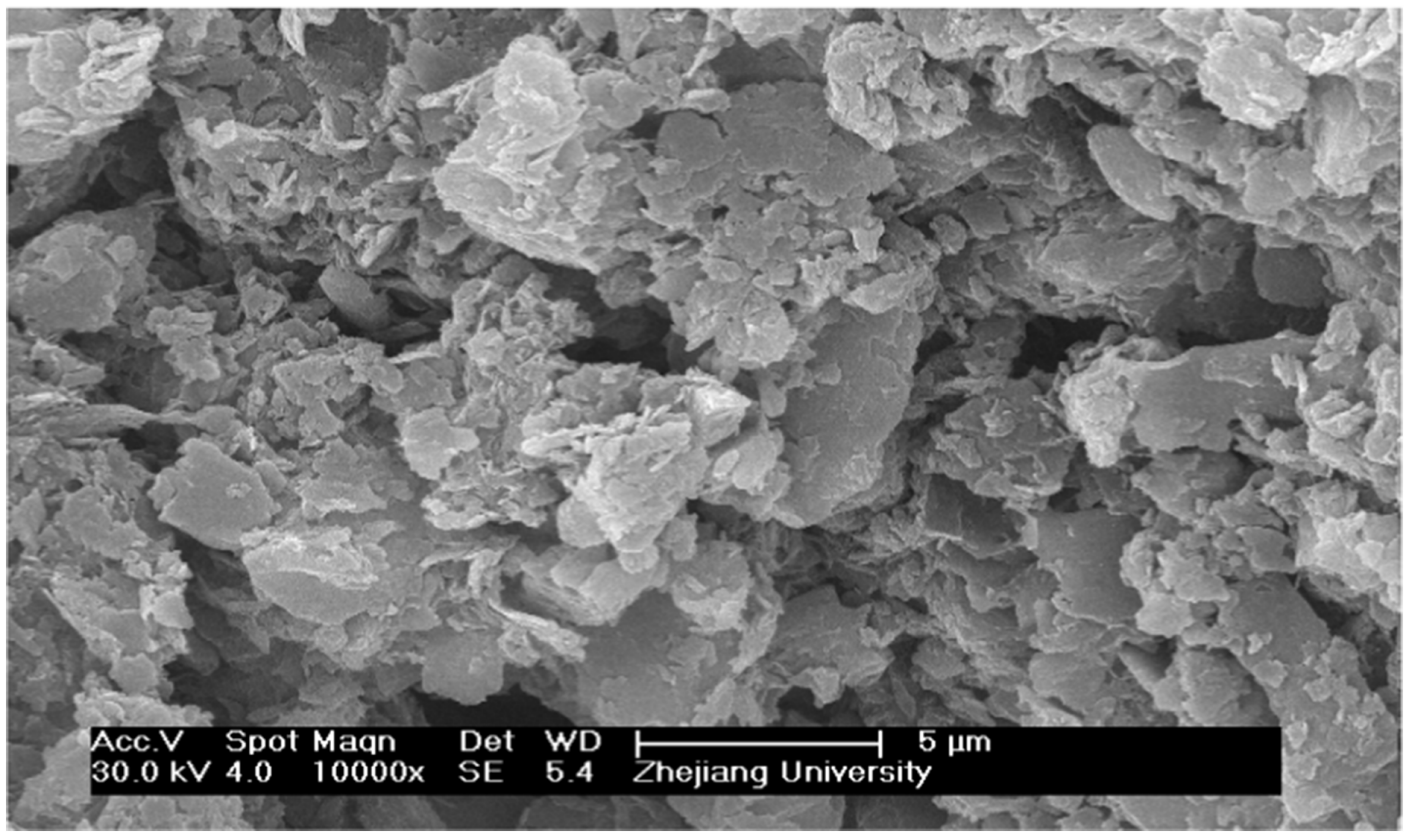
Micro structure of alum sludge from scanning electron microscopes and the magnification were 10,000 KV:25.00 TILT: 0.70 TAKE-OFF:33.97 AMPT:102.4 DETECTOR TYPE:SUTW-SAPPHIRE RESOLUTION:137.91.

**Figure 8:**
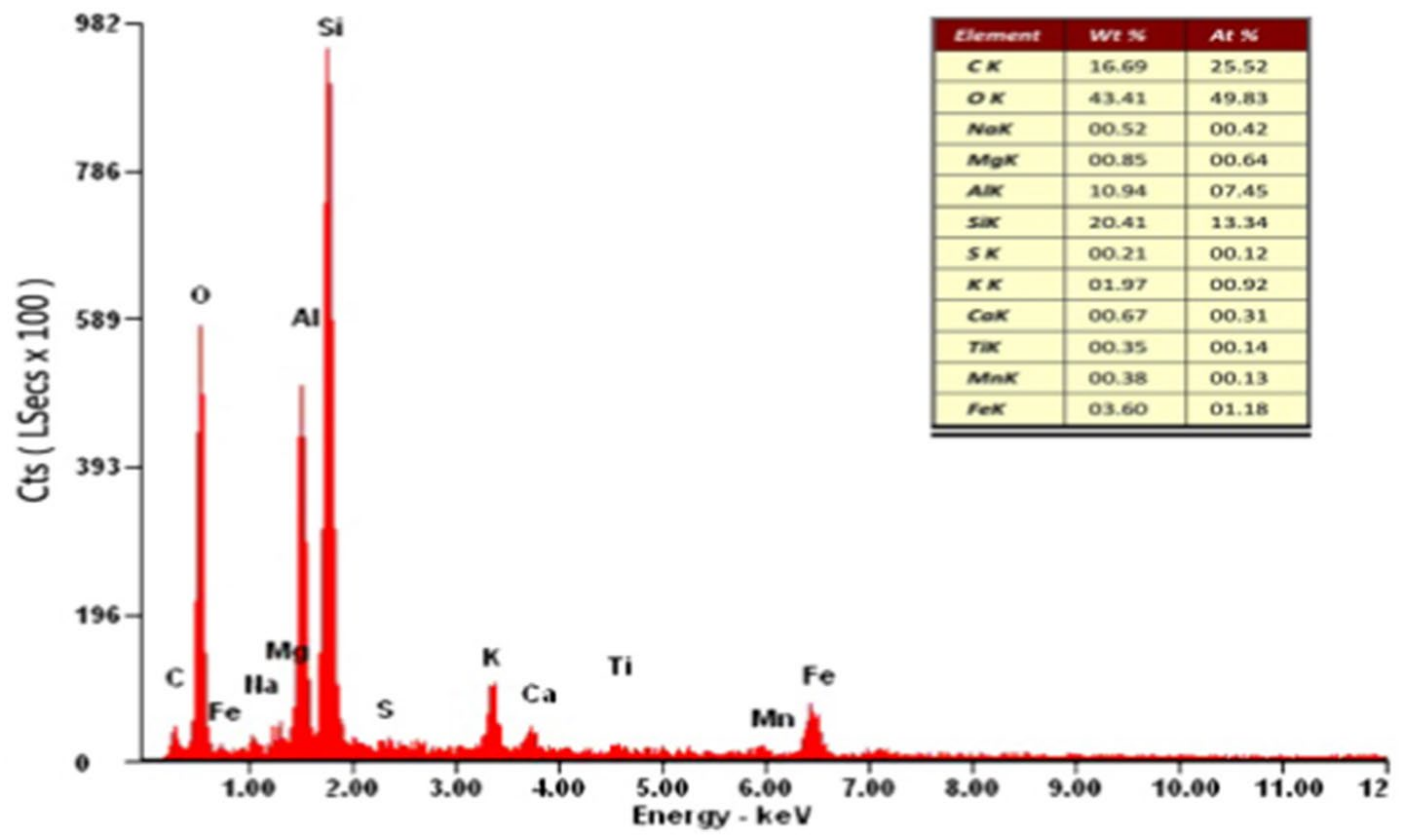
Element content analysis in Alum sludge.

**Figure 9:**
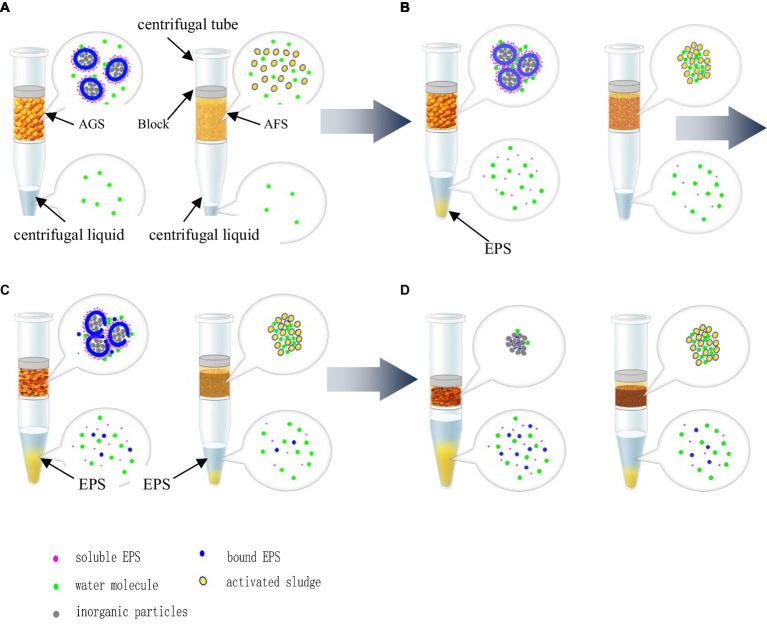
Mechanism hypothesis for AGS and AFS in centrifugal dewatering. **(A)** Centrifugal dewatering at 1 min. **(B)** Centrifugal dewatering at 5 min. **(C)** Centrifugal dewatering at 10 min. **(D)** Centrifugal dewatering at 15 min.

The scanning electron microscopy images of alum sludge was showed as Figure 7. It can be observed that the surface of the alum sludge exhibited varying degrees of roughness and possesses distinct pore structures. When mixed with activated sludge and granular sludge, these pores provided ample outflow channels for free water. According to the elemental content analysis (Figure 8), the alum sludge was primarily composed of Si and Al, accounting for 20.41 and 10.94%, respectively. It was evident that the residue from the water treatment plant contained a significant amount of inorganic sand. Under the influence of centrifugal force, they rub and compress against each other between these inorganic sand particles and the mineralized inorganic substances in the granular sludge. Under the mutual friction and pressing forces, the two inorganic minerals generated stress and compressed the cells. During the filtration process, protein-like substances can be separated from the sludge particles. Simultaneously, during the compression process, these granular inorganic substances were able to form effective water-permeable channels and polycrystalline network structures, allowing the water within the sludge cells to be fully released. In the internal structure of activated sludge, the amount of the inorganic minerals was smaller than those in granular sludge, therefore, the amount of water released was less than that of granular sludge during the centrifuge process (Figure 9). Therefore, in the initial stage of centrifugal dewatering of mature granular sludge without adding any flocculant, the removal of free water from the sludge is easier than that of ordinary activated sludge. During centrifugal dewatering of granular sludge, due to the existence of centrifugal force (Figure 9), particles can squeeze each other, and the extracellular polymers on the surface of the granular sludge are squeezed out; when a pressure block is added, the squeezing force between particles is greater, and more extracellular polymers are squeezed out, so that the water in the sludge can be fully released, and the extracellular polymers can be separated from the sludge particles on the surface of the granular sludge during the centrifugal pressure filtration process, thus further improving the dewatering performance of the granular sludge.

As a coagulant, PACL played a role in charge neutralization and adsorption bridging in sludge, which helped with the aggregation and sedimentation of sludge particles. Therefore, the residual product of PACl in alum sludge can enhance the dehydration efficiency of sludge, making it easier to perform solid–liquid separation. Additionally, the residual product of PACl in alum sludge can react chemically with organic substances in the sludge to form stable complexes, further promoting sludge dehydration. Thus, this chemical conditioning effect can reduce the content of bound water in the sludge and improve its dehydration performance.

In summary, the sludge from the water treatment plant can serve as a conditioning agent for sludge dehydration, exerting both physical and chemical conditioning effects that significantly improve the dewatering performance of the sludge. It is important to note that the dehydration mechanism of granular sludge may vary depending on the nature of the sludge, dehydration conditions, and operation methods. Therefore, in practical applications, it is necessary to select appropriate dehydration methods and operating conditions based on specific circumstances to achieve efficient and stable dehydration results.

## Conclusion

1. The specific resistance to filtration (SRF) of Aerobic Granular Sludge (AGS) was significantly lower compared to that of Activated Fine Sediment (AFS), indicating that the dewatering performance of AGS was superior to conventional AFS.
2. The centrifugal dewatering technology with addition of sludge conditioning agents could enhance the dewatering performance of AGS and AFS. the addition of alum sludge enhanced the dewatering effect.
3. AGS structure is different from AFS, results in different dewatering mechanisms and methods for them: (a) Free water could be quickly removed by filtration under gravity of AGS. (b) The removal of bound water in AGS was aided by external stress. It is recommended that the free water should be removed first by low pressure filtration, and the bound water be removed by pressure filtration in later period.

## Data availability statement

The original contributions presented in the study are included in the article material, further inquiries can be directed to the corresponding author.

## Author contributions

AY: Conceptualization, Data curation, Funding acquisition, Investigation, Methodology, Project administration, Writing – original draft, Writing – review & editing. YC: Data Curation, Investigation, Validation, Resources, Writing – review & editing. NL: Supervision, Writing – review & editing. TM: Software, Writing – review & editing. YQ: Data curation, Software, Visualization, Writing – review & editing. DX: Data curation, Investigation, Writing – review & editing.
